# Rethinking Alzheimer's: Harnessing Cannabidiol to Modulate IDO and cGAS Pathways for Neuroinflammation Control

**DOI:** 10.1523/ENEURO.0114-25.2025

**Published:** 2025-10-10

**Authors:** Sahar Emami Naeini, Bidhan Bhandari, Breanna Hill, Nayeli Perez-Morales, Hannah M. Rogers, Hesam Khodadadi, Nancy Young, Lívia Maria Maciel, Jack C. Yu, David C. Hess, John C. Morgan, Évila Lopes Salles, Lei P. Wang, Babak Baban

**Affiliations:** ^1^DCG Center for Excellence in Research, Scholarship, and Innovation (CERSI), Augusta University, Augusta, Georgia 30912; ^2^Department of Oral Biology, Dental College of Georgia, Augusta University, Augusta, Georgia 30912; ^3^College of Science and Mathematics, Augusta University, Augusta, Georgia 30912, Departments of; ^4^Neurology, Medical College of Georgia, Augusta University, Augusta, Georgia 30912; ^5^General Dentistry, Dental College of Georgia, Augusta University, Augusta, Georgia 30912; ^6^Surgery, Medical College of Georgia, Augusta University, Augusta, Georgia 30912; ^7^Georgia Institute of Cannabis Research, Medicinal Cannabis of Georgia LLC, Augusta, Georgia 30912

**Keywords:** Alzheimer's, cannabidiol, CBD, cGAS, IDO, neuroinflammation

## Abstract

Alzheimer's disease (AD) has traditionally been associated with amyloid-β plaques, but growing evidence underscores the role of neuroinflammation in disease progression. The autoinflammatory hypothesis of AD suggests chronic immune dysfunction contributes to neuronal damage, making immune modulation a promising therapeutic strategy. Cannabidiol (CBD), a phytocannabinoid with anti-inflammatory properties, may offer therapeutic potential. This study investigates how CBD independently influences two key neuroinflammatory regulators in AD: the indoleamine 2,3-dioxygenase (IDO) pathway and the cyclic GMP-AMP synthase (cGAS) pathway. Though mechanistically distinct, both shape CNS immune responses. Targeting these immune-metabolic axes provides a mechanistic alternative to amyloid- or tau-based approaches by addressing upstream drivers of neuroinflammation and immune dysregulation. Using the male 5XFAD transgenic AD mouse model, we administered CBD via inhalation and assessed IDO and cGAS expression using flow cytometry, immunofluorescence (IF), and gene expression analysis. We evaluated cytokine levels and used STRING-based bioinformatics to identify CBD-target interactions. CBD treatment significantly reduced IDO and cGAS expression, correlating with decreased proinflammatory cytokines, including TNF-α, IL-1β, and IFN-γ. Bioinformatics identified potential interactions between CBD and immune targets such as AKT1, TRPV1, and GPR55. These targets were prioritized based on their roles in neuroinflammatory signaling and high-confidence interactions with CBD. AKT1 regulates inflammatory signaling and cell survival, TRPV1 modulates nociception and neuroinflammation, and GPR55 influences immune cell activation. These findings support CBD as a potential monotherapy or adjunctive treatment for AD by targeting distinct neuroinflammatory pathways, including IDO and cGAS. Further studies are warranted to fully explore its therapeutic potential.

## Significance Statement

This study highlights the therapeutic potential of cannabidiol (CBD) in targeting neuroinflammation, a major driver of Alzheimer's disease (AD) progression. By modulating the IDO and cGAS pathways—critical regulators of CNS immune responses—CBD reduces proinflammatory cytokines and ameliorates immune dysfunction. These findings support the emerging autoinflammatory hypothesis of AD, which posits that chronic inflammation underlies neuronal damage. The IDO/cGAS signaling axis, located at the intersection of innate immunity and metabolic regulation, remains underexplored in AD and represents a key intervention point to disrupt neuroinflammatory loops. This study positions CBD as a promising mono- or adjunctive therapy and reinforces the need to consider multitargeted strategies that address upstream immune mechanisms in neurodegenerative disease.

## Introduction

Alzheimer's disease (AD) remains a major challenge for healthcare, with no effective treatment to halt or slow its progression (Yiannopoulou et al., 2019; Yiannopoulou and Papageorgiou, 2020; van der Flier et al., 2023). Current therapies, such as amyloid-targeting treatments and cognitive enhancers, only offer symptomatic relief, failing to address the root causes of neurodegeneration (Yiannopoulou and Papageorgiou, 2020; Zhang et al., 2024). This highlights a critical gap in our understanding.

The complexity of AD, driven by genetic, environmental, and immune factors, means no single theory can explain it fully. While amyloid-β (Aβ) plaques and tau tangles are central to the traditional AD model, they do not account for the disease's full scope (Morris et al., 2014; Rischel et al., 2023; Weaver, 2023). For instance, individuals with amyloid plaques may not exhibit cognitive decline, and some with severe neurodegeneration lack significant amyloid or tau pathology (Farrell et al., 2017; Weigand et al., 2020). This suggests the amyloid-tau framework is incomplete and may miss key aspects of AD's true mechanisms.

Immune system dysfunction is considered a critical and obligatory step in the progression of AD. While Aβ accumulation is a key hallmark of AD pathology, recent evidence suggests that immune dysregulation can independently drive neurodegeneration. Specifically, the activation of immune responses, including microglial and astrocytic activation, has been shown to exacerbate neuroinflammation and contribute to neuronal damage. In the 5xFAD mouse model, while Aβ deposition occurs early in the disease, the immune system's role in driving neurodegenerative processes extends beyond Aβ-related events. This aligns with the autoinflammatory theory of AD, which posits that immune dysfunction, rather than being solely a consequence of Aβ accumulation, is an early and central driver of disease progression (Meier-Stephenson et al., 2022). Therefore, immune system dysfunction is considered a central feature of AD pathology that plays a significant role in disease progression, potentially independent of the Aβ-induced toxic effects.

The autoinflammatory theory of AD (AD^2^) suggests the emerging role of indoleamine 2,3-dioxygenase (IDO) in AD progression. IDO, an enzyme involved in tryptophan metabolism, plays a dichotomic role in neuroinflammation. It can either promote immune tolerance and anti-inflammatory responses or contribute to neurotoxic inflammation (Kwidzinski and Bechmann, 2007; Baban et al., 2011; Salminen, 2022). In AD, elevated IDO activity may indicate a maladaptive immune response, amplifying neuroinflammation and accelerating neurodegeneration (Yu et al., 2015; Savonije et al., 2023). This suggests that IDO could be a central mediator of the immune dysregulation seen in AD, linking the immune system's response to the neuroinflammatory processes driving the disease.

Cytokines and innate immune sensors independently contribute to the complex neuroinflammatory milieu of AD. Interferon-gamma (IFN-γ), a Type 2 interferon predominantly secreted by activated T-cells and natural killer cells, plays a pivotal role in amplifying inflammation by upregulating IDO in the kynurenine pathway that promotes neurotoxic tryptophan metabolites. IFN-γ–mediated induction of IDO fosters a chronically proinflammatory environment and drives microglial activation, which, in turn, enhances cytokine release and sustains neuroinflammation (Smith et al., 2012; Yu et al., 2015; Ogunmokun et al., 2021; Govindarajulu et al., 2023; Savonije et al., 2023; Chen et al., 2024). Independently, the cyclic GMP-AMP synthase (cGAS) pathway serves as a cytosolic DNA sensor that activates the STING signaling cascade, culminating in robust Type 1 interferon responses and the expression of inflammatory mediators. This cGAS-STING axis has been increasingly implicated in the progression of AD through its role in sensing DNA damage and triggering maladaptive microglial activation, thereby exacerbating neuroinflammation and neuronal dysfunction (Smith et al., 2012; Govindarajulu et al., 2023; Chen et al., 2024). While these pathways are mechanistically distinct, they intersect functionally within the broader context of neuroimmune signaling. For example, IFN-γ–driven IDO expression and cGAS-STING–mediated interferon production converge on overlapping downstream inflammatory cascades. This convergence may establish a synergistic feedback loop that intensifies immune activation, sustains glial reactivity, and accelerates disease progression. Understanding these parallel and intersecting pathways offers mechanistic insight into AD pathogenesis and highlights the therapeutic potential of modulating both cytokine-dependent and nucleic acid-sensing pathways. Building on this concept, recent insights into neurodegeneration increasingly support the idea that immune-metabolic dysregulation is not merely a downstream effect but a fundamental driver of AD pathogenesis. Specifically, the cGAS-STING pathway, a cytosolic sensor of DNA damage, and the IDO-kynurenine axis converge to regulate both innate immune activation and metabolic reprogramming in the brain. Targeting this dual-pathway interface introduces a paradigm shift beyond amyloid- and tau-focused models by offering a more integrated approach to modulating chronic neuroinflammation at its source. This perspective aligns with the AD^2^ theory and further strengthens the rationale for therapeutic strategies aimed at restoring neuroimmune homeostasis through immune-metabolic intervention.

Thus, targeting IDO activity, along with cGAS modulation, could offer a novel approach for restoring immune balance in AD. By addressing both the immune dysregulation and neuroinflammatory components of the disease, therapies aimed at these pathways could provide a promising therapeutic strategy within the autoinflammatory framework of AD^2^, potentially offering disease-modifying effects beyond amyloid- and tau-focused treatments.

Several studies have demonstrated that cannabidiol (CBD) can alleviate AD symptoms and improve patient outcomes (Baban et al., 2021; Xiong and Lim, 2021; Trojan et al., 2023). CBD, a nonpsychoactive phytocannabinoid derived from cannabis, has gained attention as a promising therapeutic agent for inflammatory diseases, largely due to its anti-inflammatory properties, low toxicity, and ability to modulate various proinflammatory signals (Baban et al., 2021; Singh et al., 2023). Recent findings suggest that CBD significantly increased TREM2 expression in glial cells and reduced IL-6 levels in peripheral blood leukocytes, which were associated with improved cognitive function in a preclinical AD model (Khodadadi et al., 2021). Additionally, CBD not only enhanced astrocytic IL-33 expression, a cytokine that promotes microglial phagocytosis of Aβ and improves contextual memory, but also boosted acetylcholine production, further improving cognitive performance (Khodadadi et al., 2021). These results support the potential of CBD as a clinically safe and effective disease-modifying treatment to slow neurocognitive decline in AD.

CBD's anti-inflammatory and immunomodulatory properties may influence key immune pathways such as IDO and cGAS, which are involved in chronic inflammation and neurodegeneration. While direct evidence of CBD's interaction with these pathways is still limited, it is plausible that CBD could modulate both the IDO and cGAS pathways, offering potential therapeutic benefits for neuroinflammation in neurodegenerative diseases like AD. In this study, we aimed to investigate the association between CBD, IDO, and cGAS to uncover new insights into CBD's role in modulating immune responses and slowing neurodegeneration, ultimately contributing to more effective, disease-modifying treatments for AD.

## Materials and Methods

### Experimental design and treatment protocol

Adult male 5xFAD mice were purchased from Jackson Laboratory. The 5xFAD mice express human amyloid precursor protein and preseniln-1 transgenes with five AD-linked mutations, used as a preclinical model of AD (Oakley et al., 2006; Schneider et al., 2014). At 9–12 months, mice were randomized into two groups (*n* = 10/group), receiving either placebo or inhaled CBD (10 mg/mouse, Thriftmaster Global Bioscience) every day for 4 weeks. Each CBD inhaler contained 985 mg of broad-spectrum CBD (winterized crude hemp extract) plus 15 mg of cosolvent, surfactant, and propellant, with a total volume of 1,000 mg (1.78 mg dose per actuation, with 200 ml/min flow rate). For the placebo, the 985 mg of broad-spectrum CBD was replaced with 985 mg of hemp seed oil. As described previously (Khodadadi et al., 2024), inhalers were modified by adding an extra nozzle piece to adjust to the mouse model and to further control the intake of CBD. The study design included three independent cohorts, ensuring the robustness and reproducibility of the results. Inhalation was selected as the route of administration based on its favorable pharmacokinetic profile, including rapid systemic absorption and avoidance of first-pass hepatic metabolism, which can reduce the bioavailability of orally administered CBD (Stella et al., 2021; Varadi et al., 2023). This method allows for more consistent plasma concentrations and a faster onset of action, which is essential for capturing dynamic neuroimmune responses within a defined experimental window.

### IF staining

Fresh brain tissues were fixed with 10% neutral buffered formalin. The tissues then were processed, embedded, and subsequently cut into 4 μm sections for IF assessments as described previously (Khodadadi et al., 2021). Briefly, after blocking endogenous peroxidase activity with hydrogen peroxide (diluted 1:10 in distilled water for 10 min), sections were treated with Proteinase K for 10 min and washed twice in PBS. Slides were then labeled with specific, fluorescent-conjugated antibodies with fluorescent conjugation against IDO (PE anti-IDO1, BioLegend, catalog #654006), transmembrane protein 119 (Alexa Fluor 488 anti-TMEM119, for microglia, Thermo Fisher Scientific, catalog #53-6119-82), glial fibrillary acidic protein (Alexa Fluor 488 anti-GFAP, for astrocytes, BioLegend, catalog #644704), and cGAS (catalog #79978, purified, Cell signaling Technology). All slides were counterstained using DAPI (4′,6-diamidino-2-phenylindole, Thermo Fisher Scientific, catalog #D1306) prior to examination and imaging by Zeiss Fluorescence Microscope. The integrated density of the area chosen (in pixels) and the mean gray value (the measurement of the brightness) were measured using the lasso tool of Adobe Photoshop CS4 extended.

### Analytical flow cytometry

For flow cytometry analysis, single-cell suspension was prepared from the brain by sieving the brain tissues through a 100 μm cell strainer (BD Biosciences) followed by centrifugation (252 × *g*, 10 min). All cells were then stained with fluorescent antibodies based on routine flow cytometry staining protocol as described previously (Khodadadi et al., 2021). Briefly, all cells were then stained with fluorescent antibodies to phenotype and quantify microglia (TMEM119^+^CD45^+/lo^) and infiltrating macrophages (TMEM119^−^CD45^+/hi^; Alexa Fluor 488 anti-TMEM119, Thermo Fisher Scientific, catalog #53-6119-82 and PE anti-mouse CD45, BioLegend, catalog #157604). Then cells were fixed and permeabilized and stained intracellularly for cytokines including IFN-γ (APC anti-mouse IFN-γ, BioLegend, catalog #505809), interleukin-1β (PerCP anti-mouse IL-1β, Thermo Fisher Scientific, catalog #46-7114-82), tumor necrosis factor alpha (Pacific Blue anti-mouse TNFα, BioLegend, catalog #506318), and interleukin-10 (Biotin anti-mouse IL-10, BioLegend, catalog #505003). Cells were then run through a NovoCyte Quanteun flow cytometer (Agilent Technologies) and analyzed by the FlowJo V10 analytical software. To confirm the specificity of primary antibody binding and rule out the nonspecific Fc receptor binding to cells or other cellular protein interactions, negative control experiments were conducted using isotype controls matched to each primary antibody's host species, isotype, and conjugation format.

### Behavioral tests

Cognitive and exploratory behaviors were evaluated using the open field (OF) and novel object recognition (NOR) paradigms, adapted from established protocols (Khodadadi et al., 2024). For NOR, mice were initially placed in an enclosed arena containing two identical objects positioned within a 10 cm circular boundary at a fixed distance apart. After a familiarization phase, animals were briefly removed, and upon reintroduction, one of the familiar objects was substituted with a novel object differing in shape, texture, and appearance. Recognition memory was quantified using a discrimination index (DI), calculated as (*T*_n_ − *T*_f_) / (*T*_n_ + *T*_f_), where *T*_n_ represents time spent exploring the novel object and *T*_f_ the familiar object.

In the OF test, mice were placed individually in a square arena (40 × 40 × 40 cm) for 10 min, while the activity was recorded by an overhead camera. Locomotor parameters, including total distance traveled, average velocity, and time spent in the central zone, were quantified using the EthoVision XT software (version 17.5, Noldus Information Technology).

### Integrated bioinformatic analysis

#### Network construction: protein–protein interaction

To investigate the IDO/cGAS genes, we used the interactive database platform STRING v.11.0 (https://string-db.org/). STRING is a database of known and predicted protein–protein interactions (PPI; Szklarczyk et al., 2023). The interactions include direct (physical) and indirect (functional) associations; they stem from computational prediction, from knowledge transfer between organisms, and from interactions aggregated from other (primary) databases.

Next, the PPI network was constructed. The confidence score cutoff was set at 0.4, and other settings were set to default.

#### Gene ontology functional analysis

After identification of the IDO/cGAS targets, the Metascape platform and the Enrichr database were used to analyze their main biological processes, cellular components, and molecular functions to perform enrichment analysis (Gable et al., 2022; Szklarczyk et al., 2023). The results were visualized using biological online tools.

### Statistical analysis

For statistical analysis, Brown–Forsythe and Welch analysis of variance (ANOVA) was used to establish significance (*p* < 0.05) among groups. For tissue quantification statistical analysis, the area of expression was compared in both placebo and CBD-treated groups using two-way ANOVA, followed by post hoc Sidak testing for multiple comparison (*p* < 0.05).

## Results

### Inhaled CBD reduces IDO expression and alters glial activation in mice with AD

**Figure 1.**
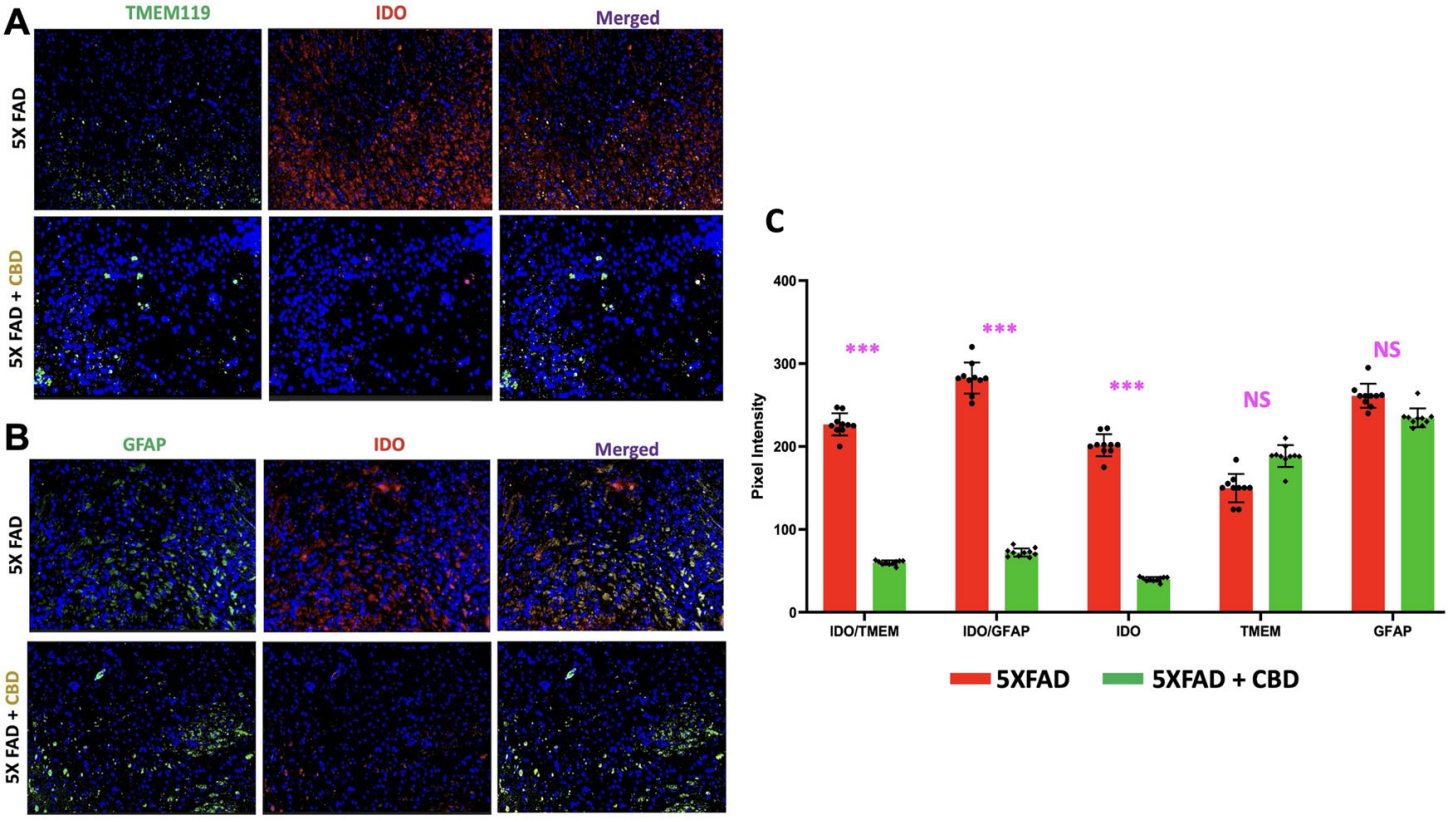
Inhalation of CBD reduces IDO expression in 5xFAD mice with AD. IF staining (Panel *A* and *B*) demonstrated a significant reduction in IDO expression in microglia (TMEM119+ cells, Panel *A*, and astrocytes (GFAP+ cells, panel *B*, within the entorhinal cortex of CBD-treated 5xFAD mice compared with untreated controls. The images shown are representative of three independent experimental cohorts. *C*, Quantitative analysis of IDO expression, based on pixel intensity measurements, confirmed a significant decrease in total IDO expression, as well as in both microglia and astrocytes, in CBD-treated mice with AD (***p < 0.001). Each graph shows all individual data points (*n* = 10 mice per group), representing one of three independent experiments with consistent findings across cohorts. Additionally, pixel intensity analysis revealed increased microglial activation (higher TMEM119 intensity) and reduced astrocyte activation (lower GFAP intensity) in CBD-treated animals relative to untreated controls, though differences in glial activation were not statistically significant (ns).

IF staining was performed to evaluate the effects of inhaled CBD on IDO expression in the brains of 5XFAD mice, a well-established model of AD. A significant reduction in IDO expression was observed in the entorhinal cortex area of both microglia and astrocytes in CBD-treated mice compared with untreated controls (Fig. 1). Specifically, in Panel A, TMEM119+ microglial cells showed a notable decrease in IDO expression following CBD treatment, while panel B demonstrated a similar reduction in IDO levels in GFAP+ astrocytes. While TMEM119 expression is known to decrease in highly activated microglia, particularly in later stages of AD, it was used here as a resident microglial marker to examine IDO expression within this population. Quantitative analysis, shown in Panel C, further confirmed these observations, with pixel intensity measurements revealing a significant decrease in total IDO expression as well as in both microglia and astrocytes in CBD-treated AD mice (***p < 0.001). In addition to these reductions in IDO expression, pixel intensity analysis indicated changes in glial activation. Specifically, CBD treatment was associated with increased microglial activation, as reflected by a higher pixel intensity of TMEM119+ staining, suggesting a heightened state of microglial response. In contrast, astrocyte activation, measured by GFAP intensity, was reduced in CBD-treated animals compared with controls, indicating a potential modulatory effect of CBD on astrocyte function. While the differences in both microglial and astrocyte activation were nonsignificant (ns), the observed trends, higher microglial activation and lower astrocyte activation, are significant in the context of AD, as increased microglial activation and reduced astrocyte activation are considered beneficial for AD progression. These changes support the potential therapeutic effects of CBD in modulating neuroinflammatory pathways in AD. The reduction in IDO expression, particularly in microglia and astrocytes, suggests that CBD may modulate key inflammatory pathways involved in AD pathology. IDO, a crucial enzyme in tryptophan metabolism, is known to be upregulated in neuroinflammatory conditions and has been implicated in neurodegenerative diseases like AD. By reducing IDO expression, CBD could potentially mitigate neuroinflammation and its contribution to AD progression, highlighting its therapeutic potential in targeting the immunoinflammatory aspects of the disease.

### CBD modulates the IDO/cGAS pathway in mice with AD

**Figure 2.**
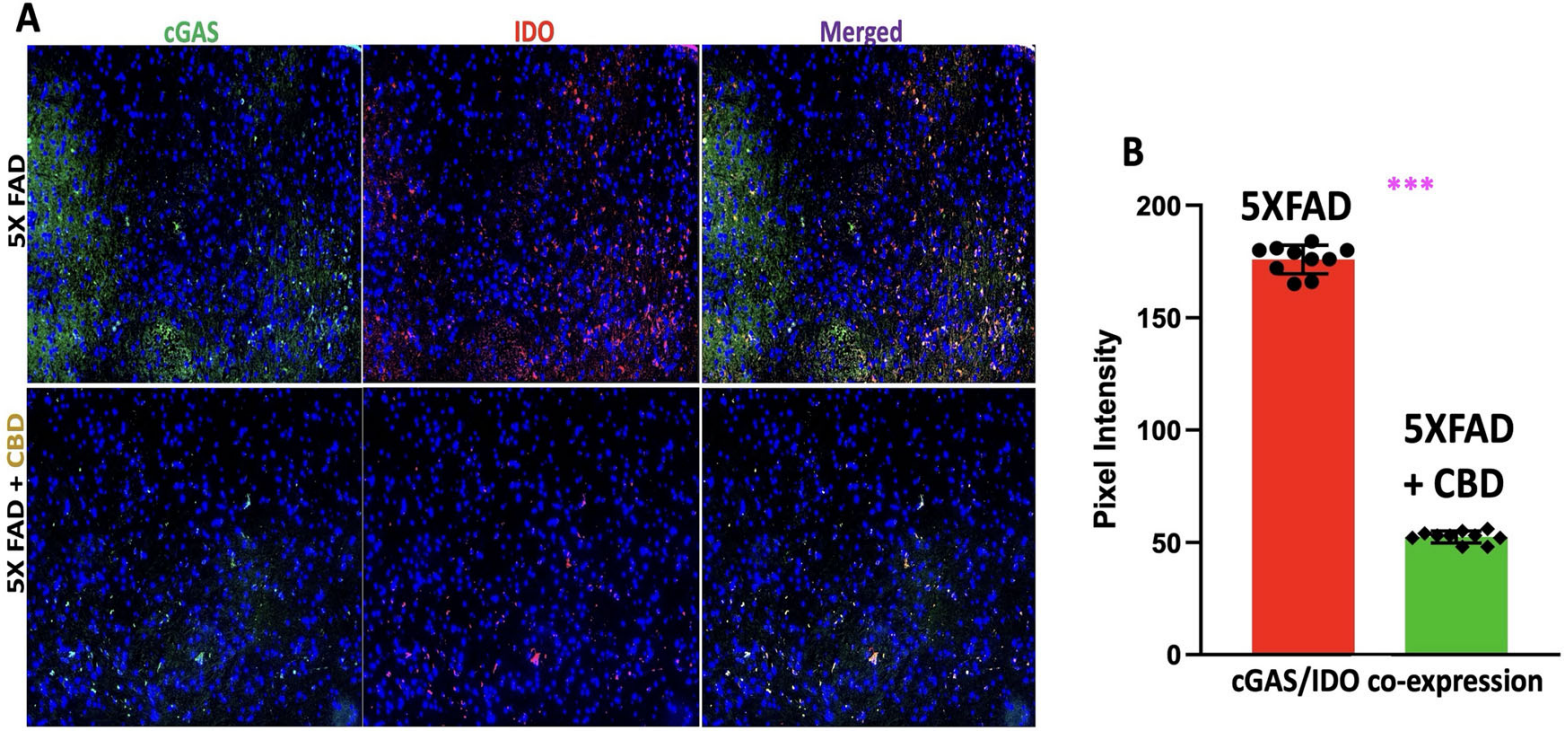
Inhalation of CBD reduces cGAS/IDO coexpression in 5xFAD mice with AD. ***A***, IF staining showed that CBD inhalation significantly reduced cGAS expression in IDO-expressing cells within the entorhinal cortex of 5xFAD mice with AD, compared with untreated controls. The images are representative of three independent experimental cohorts. ***B***, Quantitative analysis of colocalization between cGAS and IDO, based on pixel intensity measurements, revealed a significant decrease in cGAS/IDO coexpression in CBD-treated mice relative to untreated controls (***p < 0.0001). The graph displays all individual data points (*n* = 10 mice per group), derived from one representative experiment out of three biologically independent cohorts, each showing consistent results.

IF staining revealed that inhalation of CBD significantly decreased cGAS expression in IDO-expressing cells in the entorhinal cortex of 5XFAD mice with AD, compared with untreated controls (Fig. 2*A*). This reduction in cGAS expression was quantified by measuring the colocalization of cGAS and IDO, with pixel intensity analysis showing a marked decrease in cGAS/IDO coexpression in CBD-treated AD mice relative to untreated controls (Fig. 2*B*; *p* < 0.0001). These findings suggest that CBD may offer a promising therapeutic approach for AD by targeting neuroinflammatory mechanisms, specifically the IDO/cGAS pathway. This result has two key implications: First, it supports the autoinflammatory theory of AD (AD^2^), providing evidence that a more immune-targeted treatment strategy could emerge for AD, moving beyond the traditional focus on Aβ. Second, our work introduces a novel mechanism through the modulation of the IDO/cGAS pathway, which warrants further investigation into CBD's potential to alter neuroinflammation and immune responses in AD. These results underline the importance of CBD as a candidate for treating AD via immune-based pathways, expanding our understanding of its therapeutic potential in neurodegenerative diseases. We acknowledge that downstream cGAS pathway markers such as phosphorylated STING and TBK1 were not evaluated, and this remains a limitation to be addressed in future work.

### Inhaled CBD regulates proinflammatory immune profile in mice with AD

**Figure 3.**
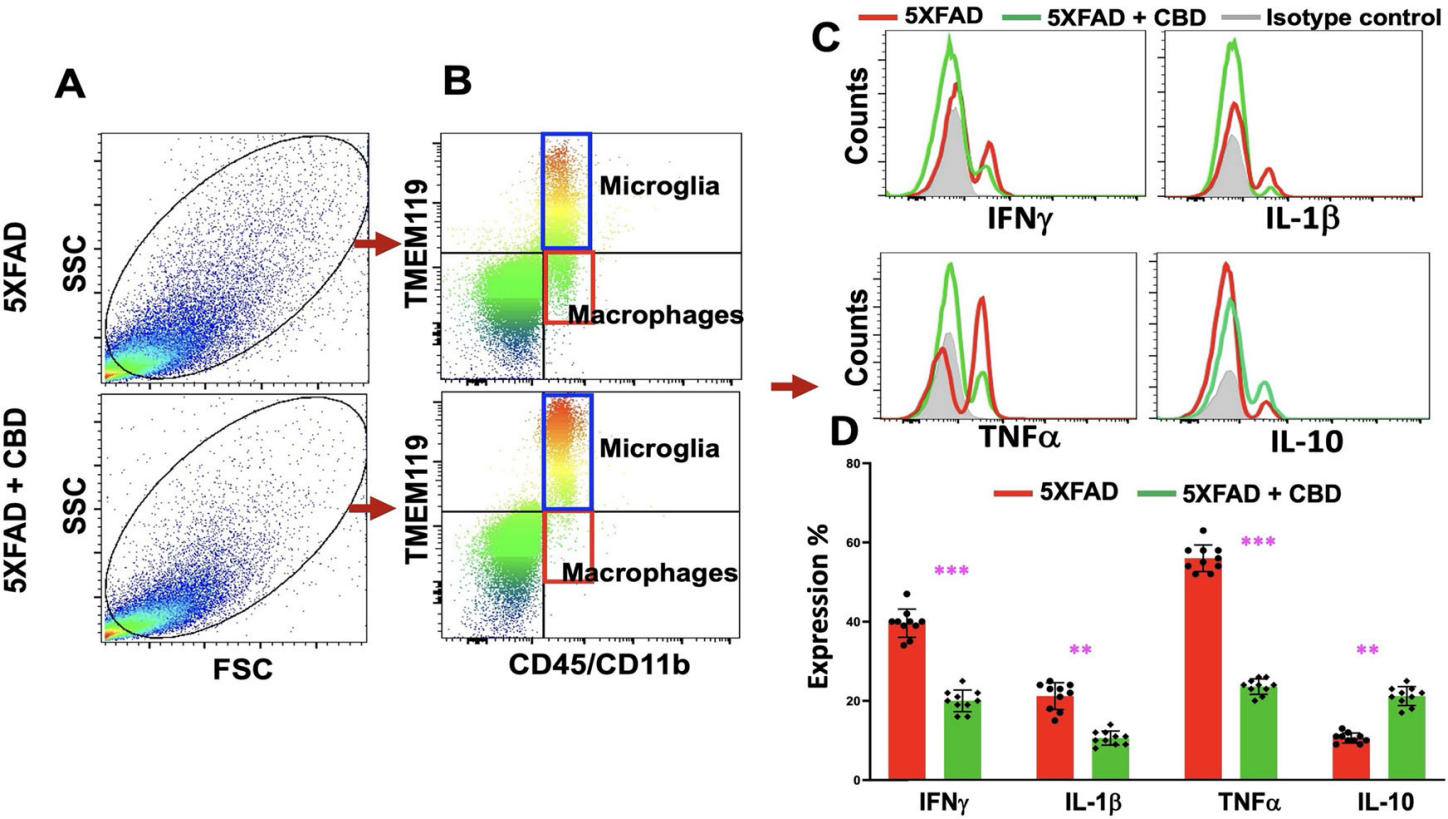
Inhaled CBD modulates the proinflammatory immune profile in mice with AD. ***A***, Flow cytometry dot plots show total brain cells from untreated (top panel) and CBD-treated (bottom panel) 5xFAD mice, with live gating based on forward scatter (FSC) and side scatter (SSC) parameters. Data are representative of three independent experimental cohorts. ***B***, Flow cytometry distinguishes resident microglia (CD45^+/lo^CD11b^+^TMEM119^+^) from infiltrating macrophages (CD45^+/hi^CD11b^+^TMEM119^−^). CBD treatment reduced the frequency of infiltrating macrophages, suggesting attenuation of inflammatory responses. ***C***, Intracellular cytokine staining revealed a significant reduction in proinflammatory cytokine production (IFN-γ, TNF-α, IL-1β) in CBD-treated mice, accompanied by enhanced production of the anti-inflammatory cytokine IL-10, relative to untreated controls. ***D***, Quantitative analysis of cytokine profiles is presented as histograms, displaying all individual data points (*n* = 10 mice per group). The dataset shown is representative of three biologically independent experiments, each yielding consistent results. Significant differences were noted between groups (**p < 0.05; ***p < 0.001).

Flow cytometry analysis was performed to assess immune cell profiles in the brains of 5XFAD mice with or without CBD treatment (Fig. 3). Panel A shows the total brain cells from untreated (top panel) and CBD-treated (bottom panel) 5XFAD mice, with live gating based on FSC/SSC parameters. Panel B reveals that CBD treatment significantly reduced the frequency of infiltrating macrophages (CD45^+/hi^CD11b^+^TMEM119^−^), a population distinguishable from resident microglia by their lack of TMEM119 expression, indicating a decrease in neuroinflammatory responses. Further analysis (Panel C) showed that CBD treatment decreased proinflammatory cytokine production while enhancing the anti-inflammatory cytokine IL-10. Panel D quantifies these changes, with significant differences (**p < 0.05; ***p < 0.001) observed in the cytokine profile. These results suggest that CBD modulates immune responses in AD by reducing macrophage infiltration and promoting an anti-inflammatory cytokine profile, supporting its potential as a therapeutic agent for neuroinflammation in AD.

### CBD treatment improves cognitive function in mice with AD

**Figure 4.**
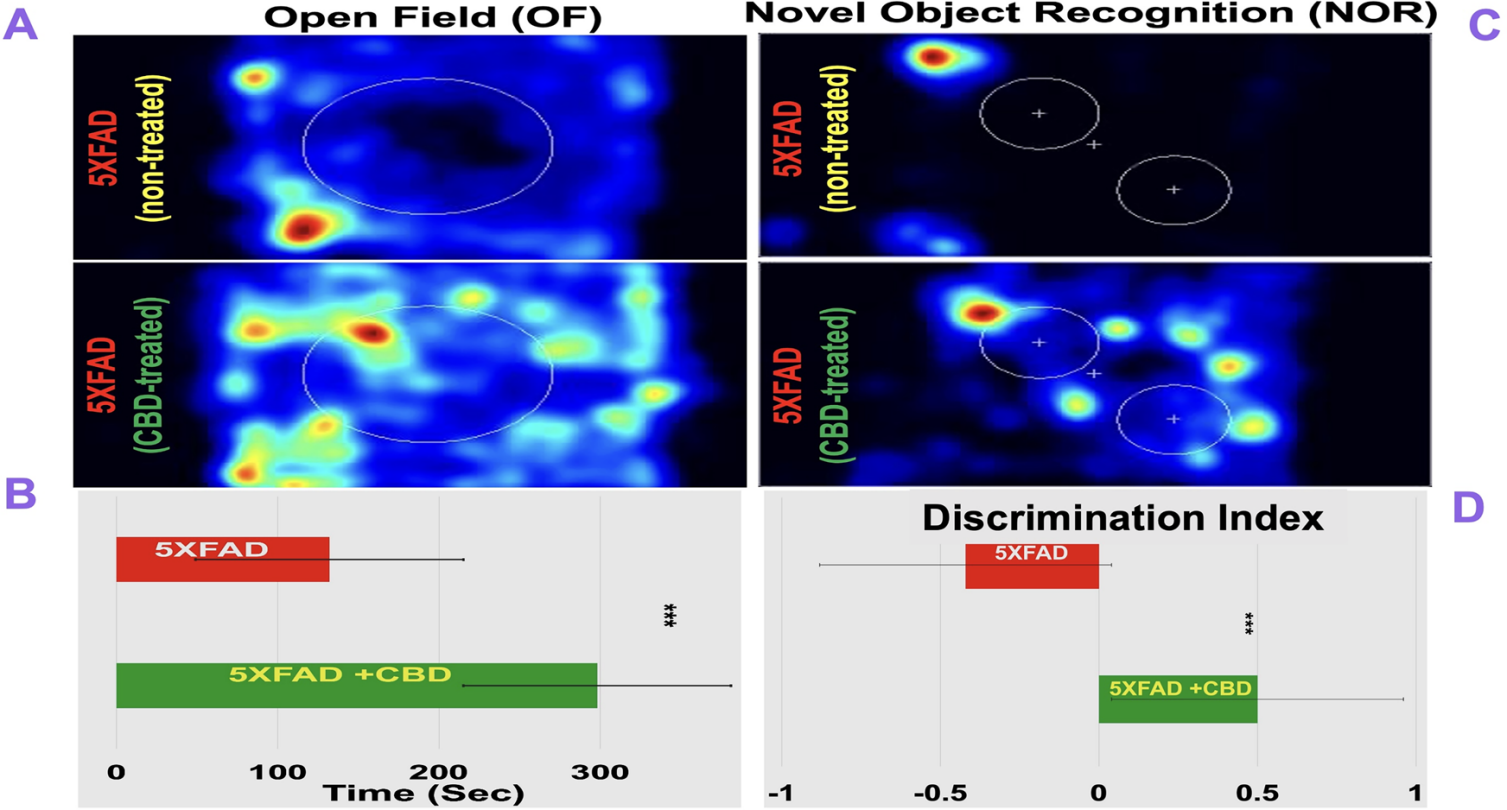
Inhaled CBD improves cognitive performance in mice with AD. ***A***, Heat maps from the OF test show increased central zone exploration in CBD-treated 5xFAD mice compared with untreated controls. ***B***, Quantification of OF performance demonstrates significantly greater time spent in the central zone for CBD-treated mice (*p* ≤ 0.001). ***C***, Heat maps from the NOR test indicate enhanced exploratory preference in CBD-treated 5xFAD mice. ***D***, DI analysis confirms improved recognition memory with CBD treatment (DI, ∼0.5 vs −0.4; *p* ≤ 0.001). Data are mean ± SEM.

To evaluate whether molecular and immunological changes induced by CBD translated into functional benefits, we assessed cognition using the OF and NOR tests. CBD-treated 5xFAD mice exhibited significantly improved behavioral performance compared with untreated controls (Fig. 4). In the OF test, CBD-treated mice spent more time in the central zone (290 vs 130 s in untreated mice; *p* ≤ 0.001), reflecting reduced anxiety-like behavior and improved exploratory activity. In the NOR test, CBD-treated mice displayed enhanced recognition memory, with a DI of 0.5 compared with −0.4 in untreated controls (*p* ≤ 0.001). Together, these findings indicate that inhaled CBD not only modulates neuroinflammatory pathways but also improves cognitive outcomes in the 5xFAD model of AD.

### Bioinformatics analysis of IDO/cGAS pathway in AD: CBD as a potential therapeutic target

**Figure 5.**
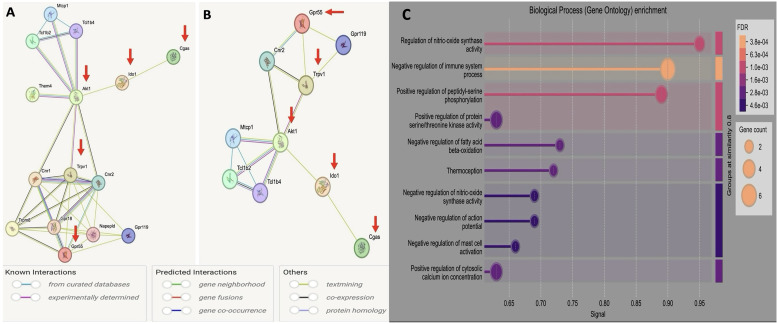
Bioinformatics analysis of the IDO/cGAS pathway in AD: CBD as a potential therapeutic target. ***A***, PPI network analysis identifies IDO and cGAS as central nodes in neuroinflammatory signaling, along with CBD-interacting targets such as AKT1, TRPV1, and GPR55. These targets were prioritized based on both their functional relevance to immune regulation and their predicted high-confidence interactions with CBD from STRING database analysis. ***B***, PPI enrichment analysis demonstrates significant coregulation between IDO/cGAS and the selected CBD-targeted nodes, suggesting coordinated involvement in neuroimmune and metabolic stress pathways implicated in AD. ***C***, Gene ontology-based pathway enrichment analysis reveals that CBD-targeted genes participate in critical biological processes including nitric oxide synthase regulation, immune system modulation, and calcium ion homeostasis, key factors that influence glial activation, cytokine signaling, and neuroprotection in the context of AD-related neuroinflammation.

To explore the potential therapeutic role of CBD in AD, we conducted a comprehensive bioinformatics analysis focused on the IDO/cGAS pathway and its molecular intersection with CBD-targeted proteins (Fig. 5). Panel A displays a STRING-derived PPI network that positions IDO1 and cGAS (MB21D1) as central hubs within a broader immune signaling architecture implicated in AD-related neuroinflammation. This network revealed multiple high-confidence interactions between immune-metabolic regulators and CBD-associated proteins. Notably, AKT1, TRPV1, and GPR55 emerged as key molecular nodes based on their connectivity within the network, their STRING confidence scores, and their established roles in regulating neuroimmune function. AKT1, a serine/threonine kinase involved in the PI3K/AKT signaling cascade, plays a pivotal role in immune tolerance, glial survival, and inflammatory cytokine regulation, functions that are dysregulated in AD and closely linked to cGAS-STING–driven interferon responses (Jia et al., 2021). TRPV1, a calcium-permeable ion channel broadly expressed in astrocytes and microglia, modulates inflammatory responses, oxidative stress, and neuroglial cross talk, thereby influencing the chronic immune activation observed in neurodegenerative contexts (Awad-Igbaria et al., 2025). GPR55, an orphan G-protein–coupled receptor increasingly recognized for its immunomodulatory properties, regulates microglial migration, cytokine secretion, and lymphocyte activation and may act as an indirect cannabinoid sensor in the CNS (Sumida et al., 2017). Panel B of Figure 5 illustrates the enriched co-regulatory relationships between these CBD-interacting proteins and the IDO/cGAS axis, reinforcing the hypothesis that CBD exerts its effects by modulating convergent neuroimmune pathways. Panel C highlights pathway enrichment data showing that the top-ranked CBD targets are involved in biological processes such as nitric oxide synthase regulation, cytokine signaling, calcium ion homeostasis, and immune effector functions, each of which plays a critical role in neuroinflammation and neuronal integrity in AD. These findings suggest that the therapeutic effects of CBD may arise from its ability to influence a coordinated immune-metabolic network involving both canonical inflammatory mediators (e.g., IDO and cGAS) and regulatory nodes such as AKT1, TRPV1, and GPR55. The inclusion of these targets was therefore driven not only by STRING-based interaction strength but also by their functional integration within pathophysiological processes central to AD progression.

## Discussion

Our study provides robust support for the evolving concept of AD as an autoinflammatory disorder (AD^2^), offering new insights into the pathophysiology of AD beyond the Aβ hypothesis (Rayaprolu et al., 2021; Weaver, 2023; Arshavsky, 2024). While amyloid plaques and tau tangles have historically been considered central to AD pathology, emerging evidence suggests that these factors alone cannot fully account for the complexity of AD progression (Naseri et al., 2019; Zhang et al., 2023; Behl, 2024). Our findings are consistent with the immune hypothesis of AD^2^, which proposes that immune system dysregulation, rather than amyloid deposition in isolation, plays a central role in the initiation and progression of neurodegenerative processes in AD (Naseri et al., 2019; Rayaprolu et al., 2021; Weaver, 2023; Zhang et al., 2023; Arshavsky, 2024; Behl, 2024). Specifically, in this model, the immune system aberrantly targets the autologous brain tissue, exacerbating neuroinflammation, a key feature of AD pathology (Latta et al., 2015; Calsolaro and Edison, 2016; Weaver, 2023). By demonstrating that CBD inhalation modulates immune-related pathways in the brain, our results provide compelling evidence supporting immune dysfunction as a major contributor to AD progression, further validating the AD^2^ model.

The choice of the 5XFAD transgenic mouse model for our studies was particularly advantageous, as it closely mimics the hallmark features of AD, including Aβ deposition, neuroinflammation, and cognitive decline (Oakley et al., 2006; Schneider et al., 2014). This model is widely recognized for its ability to reproduce key aspects of AD pathology, making it an ideal system for evaluating potential therapeutic strategies like CBD. The 5XFAD model provided a robust platform to investigate how CBD interacts with immune-related pathways in the context of AD, allowing us to generate meaningful insights into its effects on neuroinflammation and glial function.

A critical mechanism implicated in immune dysfunction within the CNS is the IDO/cGAS pathway (Lemos et al., 2020; Govindarajulu et al., 2023; Gulen et al., 2023). Both IDO and cGAS are integral to maintaining immune homeostasis in the brain (Kwidzinski and Bechmann, 2007; Baban et al., 2011; Salminen, 2022; Govindarajulu et al., 2023). IDO regulates tryptophan metabolism, and its overactivation is associated with increased neuroinflammation in various neurological conditions, including AD (Liu et al., 2018; Sodhi et al., 2021). cGAS, through its role in sensing cytosolic DNA, initiates the STING pathway, which, when dysregulated, can amplify neuroinflammation (Zhang et al., 2020; Zhang and Zhang, 2025). Our study presents novel evidence that CBD effectively modulates key components of immune regulation, highlighting its potential as a therapeutic strategy to address immune dysfunction at the core of AD pathophysiology. While our findings demonstrate that total cGAS expression is modulated by CBD treatment, we acknowledge that evaluating downstream signaling components such as phosphorylated STING (p-STING) and TBK1 (p-TBK1) would offer a more direct readout of cGAS pathway activation. However, given the novel focus of this study on the intersection of cGAS and IDO signaling as an upstream immune-metabolic axis, total cGAS expression served as a relevant marker for identifying broader immune dysregulation in AD. Future studies incorporating p-STING and p-TBK1 analysis will be essential to validate the functional activation status of this pathway and further elucidate how CBD impacts specific effector cascades downstream of cGAS. By targeting both IDO and cGAS, CBD offers a promising strategy to restore immune balance in the CNS and mitigate neurodegenerative processes. These findings underscore the emerging importance of the cGAS/IDO axis as a central regulatory node in AD-related neuroimmune dysfunction. Rather than serving merely as downstream markers of inflammation, both cGAS and IDO orchestrate fundamental immune-metabolic processes, including interferon signaling, kynurenine pathway activation, and glial reprogramming, which drive chronic neuroinflammation and neuronal injury. By modulating both DNA-sensing innate immune signaling and tryptophan catabolism, CBD exerts a dual regulatory effect that may dampen maladaptive immune activation while preserving neuroprotective immune tolerance. This layered mechanism offers a distinct therapeutic advantage over existing amyloid-centric approaches, positioning CBD as a candidate capable of targeting upstream inflammatory circuits implicated in both the onset and propagation of AD's pathology.

Our findings are in line with previous studies that have shown beneficial effects of CBD in AD models, both in mice and humans (Baban et al., 2021; Khodadadi et al., 2021; Xiong and Lim, 2021; Singh et al., 2023; Trojan et al., 2023). These studies have demonstrated that CBD's anti-inflammatory and neuroprotective properties can ameliorate key aspects of AD pathology, such as amyloid plaque deposition and cognitive impairment. Our work builds on this foundation, specifically highlighting the modulation of immune pathways like IDO/cGAS as a novel mechanism by which CBD could exert its therapeutic effects in AD. CBD's modulation of the IDO/cGAS pathway is accompanied by significant effects on glial cell activity. Our results demonstrate that CBD treatment reduces the expression of IDO in key glial populations, including microglia and astrocytes, while also decreasing the coexpression of IDO and cGAS. Given the central role of glial cells in modulating immune responses within the CNS (Wang et al., 2022), these findings suggest that CBD directly influences glial-mediated immune regulation. In conjunction with this, we also observed a CBD-induced increase in microglial activation alongside a reduction in astrocytic activation. This dual modulation is significant, as elevated microglial activation and lower astrocytic activity have been associated with enhanced neuroprotection and improved outcomes in AD (Liu et al., 2019). Chronic neuroinflammation, driven by the overactivation of immune pathways like IDO and cGAS, plays a pivotal role in AD progression (Liu et al., 2018; Zhang et al., 2020; Sodhi et al., 2021; Zhang and Zhang, 2025). Therefore, CBD's ability to regulate both glial activity and immune pathways may not only mitigate neuroinflammation but also foster a neuroprotective environment within the brain, offering a potential therapeutic benefit for AD.

Furthermore, the choice of inhalation as the route of CBD administration reflects both pharmacological and translational considerations. Inhaled delivery circumvents hepatic first-pass metabolism and provides a more rapid and reliable systemic exposure profile than oral or intraperitoneal routes (Stella et al., 2021; Varadi et al., 2023). This delivery method enhances CNS bioavailability and has shown improved efficacy in other neurological disease models. For example, a recent study in a preclinical model of epilepsy demonstrated that CBD administered via inhalation was superior to both oral and intraperitoneal routes in reducing seizure severity and neuroinflammatory responses (Bhandari et al., 2025). While our study focuses on AD, this evidence supports the broader therapeutic utility of inhalation for delivering cannabinoids in CNS-targeted interventions.

Our bioinformatic analysis provides deeper mechanistic insight into the potential pathways through which CBD may exert therapeutic effects in AD, particularly by modulating neuroimmune signaling hubs. The data suggest that CBD influences not only upstream inflammatory regulators like IDO and cGAS but also engages key molecular effectors such as AKT1, TRPV1, and GPR55, each of which plays a distinct but converging role in neuroinflammation and neuronal homeostasis. Notably, activation of the AKT1 signaling cascade by CBD may promote neuronal survival, enhance metabolic resilience, and suppress inflammatory output via PI3K-dependent mechanisms (Sun et al., 2015). This is especially relevant in the AD context, where impaired AKT1 signaling has been associated with increased vulnerability to neurodegeneration. In parallel, CBD appears to target TRPV1, a ligand-gated ion channel expressed on glial cells that regulates calcium influx and contributes to both the initiation and amplification of glial-mediated neuroinflammatory responses (Viana et al., 2022). By modulating TRPV1 activity, CBD may influence astrocyte–microglial interactions and dampen excitotoxic cascades linked to chronic neuroinflammation. Furthermore, the orphan receptor GPR55, increasingly recognized for its cannabinoid sensitivity and immune-modulatory functions, emerges as a compelling CBD-interacting target. GPR55 plays a pivotal role in leukocyte recruitment, microglial activation, and cytokine regulation within the CNS milieu, and its modulation could have broad implications for restoring immune homeostasis in AD (Viana et al., 2022). Target selection was informed by both their functional relevance to neuroimmune signaling cascades and their predicted high-affinity interaction with CBD as determined through STRING network analysis. AKT1, TRPV1, and GPR55 collectively represent mechanistically relevant and pharmacologically accessible nodes within the CBD-modulated immune network, providing a rational foundation for future studies aimed at dissecting their individual and combinatorial roles in disease modification.

The ability of CBD to modulate both immune dysfunction and neuroinflammation offers a more comprehensive approach to AD treatment compared with traditional amyloid-based therapies, which have shown limited success in clinical trials (Cummings et al., 2023). CBD's effects on the IDO/cGAS pathway, coupled with its ability to influence glial activity and neuronal survival, position it as a promising candidate for AD therapy. Moreover, CBD's impact on cytokine profiles further underscores its therapeutic potential. In our study, CBD treatment was associated with a decrease in proinflammatory cytokines, such as IFN-γ, IL-1β, and TNF-α, while increasing the production of the anti-inflammatory cytokine IL-10. This modulation of the inflammatory milieu is particularly important in AD, where chronic neuroinflammation plays a central role in disease progression (Lecca et al., 2022; C. Wang et al., 2023; M. Wang et al., 2023). By restoring immune homeostasis and attenuating neuroinflammation, CBD may help slow or halt disease progression, providing a valuable addition to current therapeutic strategies. While IFN-γ is classically associated with lymphocytes, prior studies have shown that microglia and macrophages can express IFN-γ in the inflamed or infected CNS (Suzuki et al., 2005; Kawanokuchi et al., 2006). Our findings suggest that chronic neuroinflammatory conditions in the 5XFAD model may similarly promote IFN-γ expression in these myeloid populations, potentially through cytokine-driven activation or cross talk with infiltrating immune cells. Notably, the observed reduction in IFN-γ levels following CBD treatment may reflect its capacity to reprogram innate immune responses in the brain, offering further support for its therapeutic potential in modulating aberrant neuroinflammation in AD.

The current study builds upon previously published work demonstrating that CBD treatment improves cognitive performance, reduces Aβ plaque burden, and enhances acetylcholine signaling in the 5XFAD mouse model of AD (Oakley et al., 2006; Schneider et al., 2014; Khodadadi et al., 2021, 2024). While the present work focuses primarily on the immunoregulatory effects of CBD, particularly its modulation of the IDO/cGAS axis, these mechanistic insights are conceptually grounded in our earlier findings of CBD's disease-modifying potential. Together, this body of work supports the hypothesis that neuroimmune modulation may underlie the cognitive and neuropathological improvements previously observed, thereby linking the emerging autoinflammatory framework of AD to therapeutic response.

Importantly, the observed behavioral improvements provide functional validation of our mechanistic findings. By demonstrating that inhaled CBD enhances recognition memory and exploratory behavior in 5xFAD mice, these results bridge molecular modulation of the IDO/cGAS axis with measurable cognitive benefits. This alignment between immune regulation and behavioral outcomes strengthens the translational relevance of our work, highlighting inhaled CBD as a promising strategy to simultaneously target neuroinflammation and cognitive impairment in AD.

In conclusion, our findings contribute to the growing body of evidence supporting the immune theory of AD^2^, demonstrating that CBD modulates key immune pathways, particularly the IDO/cGAS axis, which plays a critical role in immune dysregulation in AD. Our results highlight the potential of CBD as a novel therapeutic agent for AD, offering a multifaceted approach to disease management. By modulating immune responses, regulating glial activity, and promoting neuronal survival, CBD addresses several key aspects of AD pathology. These findings warrant further clinical investigation into the use of CBD, either as a monotherapy or in combination with other therapeutic strategies, to evaluate its potential for modifying the course of AD. Future work will extend this research to additional transgenic and sporadic AD models to evaluate the generalizability of these findings across diverse pathological contexts. Moreover, longitudinal studies will be essential to assess the durability and safety of chronic CBD treatment, as well as its long-term impact on cognitive decline, neuroinflammation, and neuropathological progression.

While certain methodological and biological caveats remain, as outlined in the Limitations section below, these do not diminish the overall strength of our findings, which provide novel insight into CBD's impact on neuroinflammation and neurodegeneration.

### Limitations

This study has several limitations. Only a single CBD dose was tested in 5XFAD mice; future studies should evaluate a broader range. Translation to humans remains uncertain due to species differences in metabolism and pharmacokinetics, requiring clinical trials. While our focus was on microglia and macrophages, additional immune and glial populations, including astrocytes, should be examined. Finally, reliance on TMEM119 as a microglial marker is a constraint, as its expression may be reduced in disease-associated states. Despite these caveats, the findings advance understanding of CBD's therapeutic potential in neurodegeneration.

## Synthesis

Reviewing Editor: Harald Sontheimer, University of Virginia Decisions are customarily a result of the Reviewing Editor and the peer reviewers coming together and discussing their recommendations until a consensus is reached. When revisions are invited, a fact-based synthesis statement explaining their decision and outlining what is needed to prepare a revision will be listed below. The following reviewer(s) agreed to reveal their identity: John Lukens, Shiva shahrara.

Both reviewers found your work nteresting and innovative and the focus on the AD2 theory of Alzheimer's, which highlights the role of immune dysfunction and chronic neuroinflammation in disease progression, is relevant and supports the rationale for targeting neuroinflammatory pathways in AD.

However, issues and technical shortcomings were noted that warrant revsions.

1. The manuscript should more clearly emphasize the innovative aspect of targeting the cGAS/IDO pathway in Alzheimer's. Briefly outline why this pathway is a novel target in AD and how it offers potential advantages over current therapeutic strategies.

2. The use of inhalation as the method for CBD administration needs further clarification. Please justify why this method was selected over others (e.g., oral or intraperitoneal administration), especially in the context of pharmacokinetics and therapeutic efficacy.

3. Expand on the bioinformatics analysis, particularly the selection of immune targets such as AKT1, TRPV1, and GPR55. Explain why these targets were chosen and their relevance to the neuroinflammatory processes in AD. A more detailed discussion of the interactions identified through the STRING database will also be useful.

4. The conclusion would benefit from a mention of the next steps in your research, such as investigating CBD's effects in other AD models or evaluating long-term effects. This will provide a roadmap for future studies and demonstrate the broader potential of CBD in Alzheimer's therapy.

5. The IDO and cGAS pathways are independent pathways and should be presented as such in the abstract to limit misinformation.

6. Unclear if autoimmune disease is correct as this implies that the pathology is driven by autoreactive T and B cells, which they have not shown. Autoinflammatory disease would be a more correct interpretation of their data.

7. It is unclear as to what they mean by "This aligns with the AD theory of AD" in the introduction.

8. It is strange to group IFN-gamma with cGAS in the introduction as type I IFNs, and not IFN-gamma, are downstream of cGAS activation.

9. Tmem119 is actually downregulated by activated microglia and is not a good marker to track microglial responses in AD-related disease as most activated microglia around amyloid beta pathology do not express Tmem119 in full-blown disease.

10. Changes in total cGAS expression are not as informative as evaluating downstream cGAS signaling such as p-STING and p-TBK1. It is difficult to interpret anything meaningful from their cGAS imaging data.

11. It is surprising that they see so much expression of IFN-gamma by macrophages/microglia in Figure 3 as it is widely thought that macrophages/microglia do not make IFN-gamma. Additional evidence in support of this is needed to bolster this potentially controversial claim by them.

12. To increase the rigor and reproducibility of their findings, they should present individual data points in their graphs and specifically indicate the experimental sample size in each of their experiments.

13. Without analysis of cognitive behavior, amyloid beta load, neuritic dystrophy, or neuronal loss it is difficult to ascertain whether their data interpretation is relative to disease pathogenesis.

## Author Response

Rebuttal Letter for Manuscript Revision Manuscript Title: Rethinking Alzheimer's: Harnessing Cannabidiol to Modulate IDO and cGAS Pathways for Neuroinflammation Control Journal: eNeuro, Manuscript ID# eNeuro eN-NWR-0114-25 Corresponding Author: Babak Baban, Ph.D.

Dear Editors and Reviewers, We would like to thank you for the thoughtful and constructive feedback provided on our manuscript. We appreciate the opportunity to revise and strengthen our submission. Below, we address each comment point-by-point and indicate how we have responded in the revised manuscript. Where applicable, we have indicated the locations of changes. The reviewers' comments are summarized first (in Italic), followed by our replies. All modifications made to the revised manuscript have been highlighted in YELLOW for ease of reference.

Reviewer Comment 1: The manuscript should more clearly emphasize the innovative aspect of targeting the cGAS/IDO pathway in Alzheimer's. Briefly outline why this pathway is a novel target in AD and how it offers potential advantages over current therapeutic strategies.

Response: We are in full agreement with the reviewer and appreciate the thoughtful suggestion. In response, we have revised the Abstract, Significance Statement, Introduction, and Discussion sections to more clearly emphasize the innovative aspect of targeting the cGAS/IDO axis in AD.

Reviewer Comment 2: The use of inhalation as the method for CBD administration needs further clarification. Please justify why this method was selected over others (e.g., oral or intraperitoneal administration), especially in the context of pharmacokinetics and therapeutic efficacy.

Response: We thank the reviewer for this important observation. In response, we have revised the manuscript and provided expanded justification for the use of the inhalation route in both the Methods and Discussion sections.

Reviewer Comment 3: Expand on the bioinformatics analysis, particularly the selection of immune targets such as AKT1, TRPV1, and GPR55. Explain why these targets were chosen and their relevance to the neuroinflammatory processes in AD. A more detailed discussion of the interactions identified through the STRING database will also be useful.

Response: We thank the reviewer for this valuable comment. In response, we have expanded both the Results and Discussion sections to provide a more detailed explanation of the rationale behind the selection of AKT1, TRPV1, and GPR55 as CBD-interacting immune targets. We have also elaborated on their relevance to neuroinflammatory processes in AD and provided a deeper interpretation of their interactions within the IDO/cGAS network based on STRING database analysis.

Reviewer Comment 4: The conclusion would benefit from a mention of the next steps in your research, such as investigating CBD's effects in other AD models or evaluating long-term effects.

Response: We thank the reviewer for this insightful suggestion. In response, we have revised the Conclusion section to include future research directions, including the evaluation of CBD's long-term effects and its therapeutic potential in additional AD models. These additions aim to strengthen the translational relevance of our findings and outline the next logical steps in this line of investigation.

Reviewer Comment 5: The IDO and cGAS pathways are independent pathways and should be presented as such in the abstract to limit misinformation.

Response: We thank the reviewer for this important clarification. In response, we have revised the Abstract to clearly state that the IDO and cGAS pathways are mechanistically distinct. We now explicitly note that these are independent but complementary immune pathways, both modulated by CBD in our study. The revised text aims to maintain scientific accuracy while preserving the overall flow and intent of the abstract.

Reviewer Comment 6: Unclear if autoimmune disease is correct as this implies that the pathology is driven by autoreactive T and B cells, which they have not shown. Autoinflammatory disease would be a more correct interpretation of their data.

Response: We thank the reviewer for this thoughtful clarification and have revised the manuscript to use autoinflammatory in place of autoimmune where appropriate.

Reviewer Comment 7: It is unclear as to what they mean by "This aligns with the AD theory of AD" in the introduction.

Response: We thank the reviewer for pointing this out. This was a phrasing error, and we have revised the sentence in the Introduction to clarify the intended meaning.

Reviewer Comment 8: It is strange to group IFN-gamma with cGAS in the introduction as type I IFNs, and not IFN-gamma, are downstream of cGAS activation.

Response: We thank the reviewer for this important clarification and apologize for the unintended implication. Our intent was not to categorize IFN-γ and cGAS as part of the same signaling axis, but rather to highlight their independent yet converging roles in neuroinflammation and their relevance to our study. IFN-γ was mentioned specifically for its role in inducing IDO expression, while cGAS was discussed as a separate innate immune sensor relevant to the activation of type I interferon responses. We have revised the whole segment in the Introduction to clarify this distinction and improve the accuracy of the immunological framing.

Reviewer Comment 9: Tmem119 is actually downregulated by activated microglia and is not a good marker to track microglial responses in AD-related disease as most activated microglia around amyloid beta pathology do not express Tmem119 in full-blown disease.

Response: We thank the reviewer for this important observation. We acknowledge that TMEM119 expression can be downregulated in highly activated microglia, particularly in proximity to amyloid plaques during late-stage AD. In our study, TMEM119 was used primarily to identify resident microglia populations for IDO co-localization rather than as a definitive marker of activation state. We have now clarified this point in the manuscript and have added a note in the limitations section.

Reviewer Comment 10: Changes in total cGAS expression are not as informative as evaluating downstream cGAS signaling such as p-STING and p-TBK1. It is difficult to interpret anything meaningful from their cGAS imaging data.

Response: We thank the reviewer for this thoughtful and constructive comment. We agree that downstream signaling components such as phosphorylated STING (p-STING) and TBK1 (p-TBK1) would provide a more direct and comprehensive assessment of cGAS pathway activation. In this study, our primary objective was to examine total cGAS expression in conjunction with IDO, highlighting what we believe to be a novel intersection of innate immune sensing and metabolic regulation in the context of AD. Our findings suggest that total cGAS expression is modulated by CBD treatment and may reflect upstream immune dysregulation. Nevertheless, we fully acknowledge the importance of evaluating downstream effectors of the cGAS-STING axis in future work to better define functional outcomes. We have added a statement in the Discussion to clarify this point and emphasize the need for further mechanistic investigation.

Reviewer Comment 11: It is surprising that they see so much expression of IFN-gamma by macrophages/microglia in Figure 3 as it is widely thought that macrophages/microglia do not make IFN- gamma. Additional evidence in support of this is needed to bolster this potentially controversial claim by them.

Response: We thank the reviewer for this excellent and insightful comment. We agree that it is not typical for microglia and macrophages to be major sources of IFN-γ under physiological conditions. However, under certain pathological and inflammatory states, including infection and neurodegenerative disease, there is evidence that these myeloid populations can express and produce IFN-γ within the CNS. Previous studies have demonstrated IFN-γ production by microglia and macrophages in the brain in response to inflammatory stimuli. For instance, Kawanokuchi et al. (2006) showed that activated microglia can express IFN-γ in vitro and in vivo in models of multiple sclerosis. Similarly, Suzuki et al. (2005) reported that both microglia and macrophages contribute to innate IFN- γ production following Toxoplasma gondii infection in the brain. In our AD model, it is possible that chronic neuroinflammation or amyloid-driven immune priming induces a heightened activation state in microglia and infiltrating macrophages, permitting expression of cytokines typically associated with lymphoid populations. Potential mechanisms may include local exposure to pro-inflammatory cytokines such as IL-12 and IL-18, known inducers of IFN-γ in myeloid cells under inflammatory conditions, or cross-talk with infiltrating lymphocytes within the neuroinflammatory milieu. While we acknowledge that additional functional validation would help strengthen this observation, we believe our findings align with emerging evidence that myeloid cells in the CNS may exhibit unexpected cytokine profiles in chronic neuroinflammatory settings. We have clarified this point in the Discussion and included supporting references to ensure appropriate interpretation of this result.

Reviewer Comment 12: To increase the rigor and reproducibility of their findings, they should present individual data points in their graphs and specifically indicate the experimental sample size in each of their experiments.

Response: We have revised all figure panels to include individual data points and added specific sample sizes to each figure legend.

Reviewer Comment 13: Without analysis of cognitive behavior, amyloid beta load, neuritic dystrophy, or neuronal loss it is difficult to ascertain whether their data interpretation is relative to disease pathogenesis.

Response: We thank the reviewer for this thoughtful and important comment. We fully agree that correlating molecular and immunological changes with cognitive and neuropathological outcomes is essential for establishing disease relevance in Alzheimer's disease. The present study was designed to build upon previously published work demonstrating that cannabidiol (CBD) improves cognitive performance, reduces amyloid-beta plaque burden, and enhances cholinergic signaling in transgenic AD models.

In the current manuscript, our aim is to extend these findings by investigating the immunological mechanisms underlying CBD's actions, with a particular focus on modulation of the IDO/cGAS pathway within the context of an emerging autoinflammatory framework. To clarify the continuity between behavioral/pathological outcomes and the mechanistic insights presented here, we have added a statement at the end of the Discussion section to explicitly highlight how this work advances the field by connecting immunomodulatory effects of CBD with previously characterized disease-modifying outcomes.

We appreciate the opportunity to strengthen the contextual framing of the current study and thank the reviewer for prompting this clarification.

We thank the reviewers once again for their detailed and insightful feedback, which has greatly improved the clarity and rigor of our manuscript.
